# Metformin reverses mesenchymal phenotype of primary breast cancer cells through STAT3/NF-κB pathways

**DOI:** 10.1186/s12885-019-5945-1

**Published:** 2019-07-23

**Authors:** José Esparza-López, Juan Francisco Alvarado-Muñoz, Elizabeth Escobar-Arriaga, Alfredo Ulloa-Aguirre, María de Jesús Ibarra-Sánchez

**Affiliations:** 1 Red de Apoyo a la Investigación (RAI), Universidad Nacional Autónoma de México- Instituto Nacional de Ciencias Médicas y Nutrición Salvador Zubirán, Vasco de Quiroga 15, Col. Belisario Domínguez Sección XVI, Delegación Tlalpan, 14080 Mexico City, CP Mexico; 2 Unidad de Bioquímica, Instituto Nacional de Ciencias Médicas y Nutrición, Salvador Zubirán Vasco de Quiroga 15, Col. Belisario Domínguez Sección XVI, Delegación Tlalpan, 14080 Mexico City, CP Mexico; 3 Hospital Ángeles del Pedregal, Camino a Santa Teresa # 1055, Col. Héroes de Padierna, 10700 Mexico City, CP Mexico

**Keywords:** Breast Cancer, Epithelial-mesenchymal transition, Metformin, STAT3, NF-κB, AMPK

## Abstract

**Background:**

Breast cancer currently is the most frequently diagnosed neoplasm and the leading cause of death from cancer in women worldwide, which is mainly due to metastatic disease. Increasing our understanding of the molecular mechanisms leading to metastasis might thus improve the pharmacological management of the disease. Epithelial-mesenchymal transition (EMT) is a key factor that plays a major role in tumor metastasis. Some pro-inflammatory cytokines, like IL-6, have been shown to stimulate phenotypes consistent with EMT in transformed epithelial cells as well as in carcinoma cell lines. Since the EMT is one of the crucial steps for metastasis, we studied the effects of metformin (MTF) on EMT.

**Methods:**

Cytotoxic effect of MTF was evaluated in eight primary breast cancer cell cultures by crystal violet assay. EMT markers and downstream signaling molecules were measured by Western blot. The effect of MTF on cell proliferation and cell migration were analyzed by MTT and Boyden chamber assays respectively.

**Results:**

We observed that the response of cultured breast cancer primary cells to MTF varied; mesenchymal cells were resistant to 10 mM MTF and expressed Vimentin and SNAIL, which are associated with a mesenchymal phenotype, whereas epithelial cells were sensitive to this MTF dose, and expressed E-cadherin but not mesenchymal markers. Further, exposure of mesenchymal cells to MTF down-regulated both Vimentin and SNAIL as well as cell proliferation, but not cell migration. In an in vitro IL-6-induced EMT assay, primary breast cancer cells showing an epithelial phenotype underwent EMT upon exposure to IL-6, with concomitant activation of STAT3 and NF-κB; addition of MTF to IL-6-induced EMT reversed the expression of the mesenchymal markers Vimentin and SNAIL, decreased pSTAT3 Y705 and pNF-κB S536 and increased E-cadherin. In addition, downregulation of STAT3·activation was dependent on AMPK, but not NF-κB phosphorylation. Further, MTF inhibited cell proliferation and migration stimulated by IL-6.

**Conclusion:**

These results suggest that MTF inhibits IL-6-induced EMT, cell proliferation, and migration of primary breast cancer cells by preventing the activation of STAT3 and NF-κB. STAT3 inactivation occurs through AMPK, but not NF-κB.

**Electronic supplementary material:**

The online version of this article (10.1186/s12885-019-5945-1) contains supplementary material, which is available to authorized users.

## Background

Breast cancer is a major health problem in women worldwide, with an estimated 1.7 million women diagnosed with this neoplasia in 2012 [1]. Approximately 30% of breast cancer patients will eventually develop metastatic disease, which is the main cause of death, particularly when present at distant organs. Currently, predicting accurately the risk for metastasis in a particular patient is not yet feasible. In fact, more than 80% of breast cancer patients receive adjuvant chemotherapy and approximately 40% will relapse and eventually die from metastatic disease. According to the widely held model of metastasis, rare subpopulations of cells within the primary tumor acquire advantageous genetic alterations over time, thereby enabling these cells to metastasize and form new solid tumors at distant sites [2]. Thus, increasing our understanding on the molecular mechanisms leading to metastasis might improve the clinical and pharmacological management of the disease.

The epithelial-mesenchymal transition (EMT) plays a major role in tumor progression by assisting invasion and intravasation of neoplastic cells into the bloodstream and inducing proteases involved in the degradation of the extracellular matrix (ECM) [3]. During the EMT, cell-cell junctions and cell adhesion to ECM are lost and, concomitantly, the apical-basolateral polarity is disrupted, enabling the cells to evolve into a mesenchymal phenotype with invasive properties [4]. Down-regulation of E-cadherin has been reported to reflect progression and metastasis in breast cancer associated with poor prognosis [5, 6]. In addition, both down-regulation of E-cadherin and up-regulation of Vimentin and N-cadherin are frequently observed in cancer cells from epithelial cancers during stromal invasion [7]. Down-regulation of E-cadherin is believed to result in loss of adhesion between epithelial breast cancer cells and other epithelial cells, whereas N-cadherin increase promotes adhesion and intrusion of tumor cells into the stroma [8]. Studying EMT in vitro has facilitated the characterization of the several signaling pathways typically involving a series of genes proposed as “EMT master genes”. These genes are a group of transcription factors that include SNAIL, TWIST, ZEB and E47 [9]. Extrinsic signals from soluble mediators from the tumor microenvironment have been implicated in the regulation of EMT.

Some cytokines have been shown to stimulate phenotypes consistent with EMT in transformed epithelial as well as carcinoma cell lines. One of these is IL-6, a pleiotropic cytokine that participates in acute inflammation, and that also plays a central role in hematopoiesis, tumor progression, and proliferation; in addition, this cytokine has been found within the tumor microenvironment [10–12]. IL-6 signaling uses a specific IL-6 receptor (IL-6R/CD126) as well as a common transmembrane signal transducer, gp130 (CD130) to initiate the JAK/STAT3 and NF-κB signaling pathways. In fact, elevated serum levels of IL-6 have been associated with poor prognosis of lung and breast cancer [13–15]. Several studies have found that IL-6 contributes to the induction of EMT in several types of tumors including lung, head and neck, breast, and ovarian cancers [16–19].

Since the EMT is one of the crucial steps for metastasis, there is an enormous interest to find strategies aimed to interrupt this process and to establish new strategies for cancer treatment. Metformin (MTF), an anti-diabetic drug widely prescribed for treating type 2-diabetes, has been associated with reduction in the risk to develop distinct types of cancer [20–22]. Several signaling pathways have been reported as putative mechanisms involved in the anti-tumor function of MTF, including inhibition of pro-inflammatory cytokines similar to IL-6 [23] and down-regulation of EMT markers such as E-cadherin, TWIST, ZEB, and Slug [24]. In lung adenocarcinoma cells, MTF has been shown to affect IL-6-induced EMT, most likely through inhibition of STAT3 phosphorylation [25]. Some anticancer effects of MTF have been associated with activation of adenosine monophosphate protein kinase (AMPK). AMPK is an energy sensor that is activated under low glucose levels, hypoxia and stress [26]. To overcome a stress condition, AMPK limits anabolic processes and activates catabolic processes to generate energy, thereby increasing cell survival under stress [27]. Another mechanism of action proposed for the MTF effects on tumor cells is through inhibition of the electron transport chain of the mitochondria, hence decreasing Complex I activity of the respiratory chain and the oxidative phosphorylation of cells [28, 29]. Moreover, inhibition of Complex I lowers the ATP production, leading to increase ADP levels that later are converted to AMP, ultimately activating AMPK [30, 31].

In the present study, we used a model of cultured primary breast cancer cells to analyze the impact of MTF on the EMT. We employed patient-derived breast cancer cell models because they represent better the molecular characteristics from the original tumors and these models are clinically relevant. We used 2 groups of primary breast cancer cells, a group with mesenchymal phenotype and another with epithelial phenotype. We found that the response to MTF is different between mesenchymal and epithelial primary breast cancer cells. MTF can suppressed basal mesenchymal markers with reduction of cell proliferation, but it did not modify cell migration rate. Furthermore, in an IL-6-induced EMT model, MTF diminished IL-6-induced cell proliferation, and migration by reducing the phosphorylation of STAT3- and NF-κB. Moreover, inhibition of STAT3 activation by MTF appeared to be dependent on AMPK activation, but not on the reduction of NF-κB phosphorylation.

## Methods

### Antibodies and reagents

Recombinant human IL-6 was purchased from PeproTech (Rocky Hill, NJ, USA). E-cadherin and Vimentin antibodies were obtained from GeneTex (Irvine, CA, USA). SNAIL, pNF-κB-p65 (Ser536), pAMPK (Thr172), AMPK, GAPDH were purchased from Cell Signaling Technology (Danvers, MA, USA). STAT3, pSTAT3 Y705, NF-κB-p65, and β-actin were obtained from Santa Cruz Biotechnology (Dallas, TX, USA).

### Cell culture

The primary cell cultures MBCDF, MBCD3, MBCD4, MBCD17, MBCD23, MBCD25, were derived from biopsies of mastectomies performed on patients with breast cancer. The study was approved by the Ethics and Research Committee of the Instituto Nacional de Ciencias Médicas y Nutrición Salvador Zubirán (Ref. 1549, BQ0–008-06 / 9–1) as described before [32, 33]. MBCDF-D5 and MBCDF-B3 are subpopulations from the primary culture MBCDF previously characterized by Esparza-López *et. al.* [33]. Cell cultures were maintained in RPMI-1640 medium supplemented with 10% fetal bovine serum (FBS), antibiotic and antimycotic (Invitrogen Corporation, Camarillo, CA) at 37 °C in a humidified atmosphere with 5% CO_2_.

### Cytotoxicity assay

Primary breast cancer cells were seeded at a density of 7500 cells/cm^2^ in 48-well plates. MTF (MP Biomedicals, Burlingame, CA) was added at increasing concentrations (0, 0.5, 1, 5, 10, 25, 50 and 100 mM), in triplicate incubations, and incubated for 48 h. Cell viability was evaluated using the crystal violet technique. Thereafter, cells were fixed with 1.1% glutaraldehyde in PBS for 20 min, followed by staining with 0.05% crystal violet and dissolved in 10% acetic acid before measuring the absorbance at 570 nm using an ELISA plate reader. The results are expressed as the percentage of viability calculated from the absorbance of a given MTF concentration with respect to the untreated control.

### Cell stimulation

Primary breast cancer cells (MBCDF-D5, MBCD3, MBCDF-B3, MBCD23) were treated with 10 mM MTF to evaluate its effect on mesenchymal markers. MBCDF, MBCD17 were induced to EMT by adding IL-6 40 ng/mL. Cells were collected for protein extraction at day 0, 1, and 2. To induce mesenchymal-epithelial transition (MET), MBCDF and MBCD17 were treated with four different conditions: no treatment, 40 ng/mL IL-6, 10 mM MTF and the combination IL-6 + MTF. At day 0, an initial IL-6 treatment was given for 24 h. Then, MTF was added with an additional dose of 40 ng/mL IL-6 to sustain EMT. These conditions were maintained for further 24 h and cells were collected for protein extraction. For inhibition of AMPK in MBCDF and MBCD17 cells, 10 μM compound C (Dorsomorphin) was added 2 h before the addition of IL-6. To activate AMPK, MBCDF and MBCD17 cells were treated with 1 mM AICAR 2 h before adding IL-6.

### Western blot

Stimulated cultured primary breast cancer cells were lysed in a buffer containing 50 mM HEPES pH 7.4, 1 mM EDTA, 250 mM NaCl, 1% Nonidet P-40, 10 mM NaF, and 1X protease inhibitors (Complete EDTA-free, Roche). Twenty micrograms of whole cell lysate were subjected to SDS-PAGE and transferred to an Immobilon-P PVDF membrane (Millipore Corp. Bedford, MA). The membrane was blocked for 60 min in 5% non-fat milk in PBS-Tween and then incubated with the corresponding primary antibodies overnight at 4 °C and thereafter with secondary anti-mouse-HRP or anti-rabbit-HRP antibodies (Jackson Immuno-Research, West Grove, PA, USA). Detection of the HRP signal was performed using the ECL™ Prime Western Blotting Detection Reagent (GE Healthcare, Buckinghamshire, UK). Blot images were digitized using Chemidoc (Bio-Rad, Hercules, CA, USA).

### Cell proliferation

Cell proliferation of cultured primary breast cancer cells in the presence of 10 ng/mL IL-6, 10 mM MTF or IL-6 + MTF was assessed by seeding 2500 cells/cm^2^ (5000 cells/well) in 24-well plates in RPMI 1640 supplemented with 10% FBS. Cell proliferation was analyzed by the MTT assay (3-[4,5-dimethylthiazol-2-yl]-2,5-diphenyl-tetrazolium bromide, Sigma-Aldrich, St Louis, MO, USA) at 0, 1, 3 and 5 days. MBCDF-D5, MBCD3, MBCDF-B3 and MBD23 cells were plated at the same density as above. Cell proliferation was evaluated after addition of MTF 0, 5, 10 and 25 mM on day 0 and 5 by MTT assay. Formazan salt was dissolved with acidulated isopropanol. The absorbance was read at 530 nm and 630 nm in an ELISA reader. Results are expressed as the increase in absorbance (570–630 nm) at days 1,3 and 5 over the absorbance (570–630 nm) on day 0. The experiments were repeated at least three times in triplicate incubations.

### Migration assay

Cell migration of MBCDF and MBCD17 cells was carried out using a Boyden chamber assay. The upper chamber was sown with 30,000-cells/200 μl in RPMI 1640 plus 10% of FBS. The lower chamber contained the following conditions: control (no additions), 10 ng/mL IL-6, 10 mM MTF, or 10 ng/mL IL-6 plus 10 mM MTF. In the case of MBCDF-D5, MBCD3, MBCDF-B3, and MBCD23 cells were seeded at the same density as above. MTF was added in the upper and lower chamber at 0. 5, 10, and 25 mM. In all conditions, cells were incubated for 6 h at 37 °C and 5% CO_2_. Non-migrating cells were removed from the upper chamber with a cotton swap. The migrating cells on the Boyden chamber were fixed with 1.1% glutaraldehyde in PBS for 20 min and then stained with crystal violet for 20 min. Cells were then counted from five random fields. The number of migrating cells was obtained by dividing the mean of the 5 fields counted by 0.001cm^2^ (viewing field area) and then multiplied by the insert area (0.33 cm^2^).

### Statistical analysis

Data are presented as mean ± SEM of three independent experiments. MTF dose-response curves were analyzed by Student’s t-test using SPSS 22.0. ANOVA was applied to proliferation and migration assays and multiple comparisons were then performed employing the Turkey HSD post-hoc test using GraphPad PRISM v6.01. *P* < 0.05 was considered significant.

## Results

### Primary breast cancer cells present variable responses to metformin

**Fig. 1:**
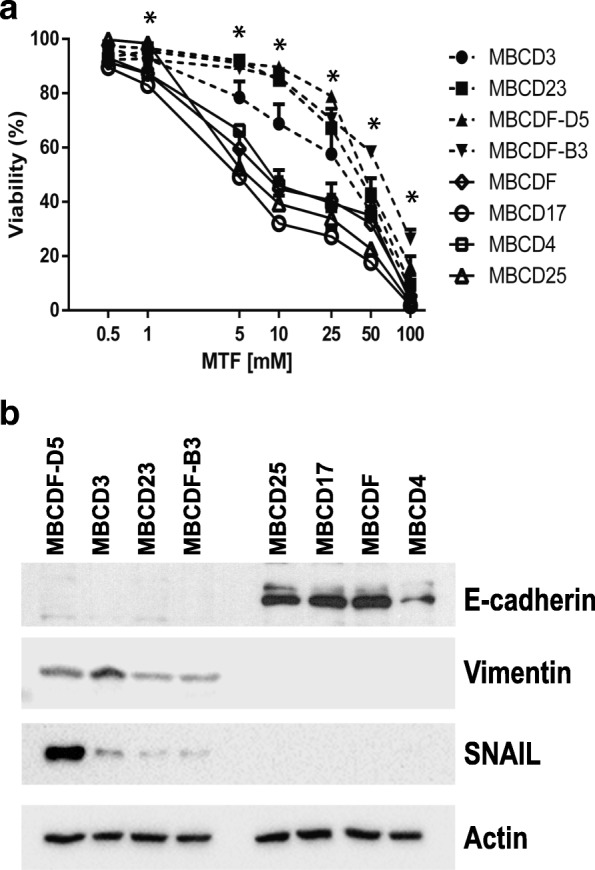
*Metformin-resistance correlates with mesenchymal phenotype in primary breast cancer cells.* **a** MBCD3, MBCD23, MBCD-D5, MBCD-B3, MBCDF, MBCD17, MBCD25 primary breast cancer cell were treated with increasing concentrations of MTF and cell viability was analyzed by crystal violet after 48 h. Data represent the mean ± SEM of three independent experiments in triplicate incubations. *P < 0.001 epithelial versus mesenchymal from 1 to 100 mM MTF. **b** Representative immunoblot showing E-cadherin, Vimentin, and SNAIL EMT markers expression. Actin was used as loading control

For this study, we used a model of primary breast cancer cells derived from patients with this type of cancer. The molecular subtype of MBCDF-D5, MBCD3, MBCD23, MBCDF-B3, MBCD25, MBCD17, MBCDF and MBCD4 breast cancer cells was determined according to the expression of estrogen and progesterone receptors and HER2 (epidermal growth factor receptor 2) (Additional file 1: Table S1) [33], and the response to MTF in these primary breast cancer cell cultures was evaluated after treatment with increasing doses of MTF (0.5, 1, 5, 10, 25, 50, and 100 mM). We found that these cells were distributed in two groups according to their sensitivity to MTF. At low concentrations of MTF, cell viability did not show any significant difference among all breast cancer cells. The major change was observed at 5, 10, and 25 mM of MTF, where MBCDF-D5, MBCD3, MBCD23, and MBCDF-B3 cells were less sensitive to MTF. Cell viability varied from 92 to 68% at 5 and 10 mM MTF doses respectively, whereas at 25 mM MTF cell viability oscillated between 79 and 57%. MBCD25, MBCD17, MBCDF, and MBCD4 cells were more sensitive to MTF; in these cells, viability varied from 66 and 27% at the range of 5 to 25 mM MTF (Fig. 1a). To further study the difference in the response to MTF among the primary breast cancer cells used, we calculated the half inhibitory concentration (IC50) for each primary culture. The IC50 of MBCDF-D5, MBCD3, MBCD23 and MBCDF-B3 cells varied from 23.97 mM to 52.61 mM, while MBCD25, MBCD17, MBCDF, and MBCD4 cells exhibited IC50s from 5.31 to 11.45 mM (Table 1).

**Table 1:** Metformin IC50 values

Primary breast cancer cell culture	IC50 [mM]
MBCDF-D5	44.70 ± 1.06
MBCD3	23.97 ± 1.97
MBCD23	36.55 ± 1.07
MBCDF-B3	52.61 ± 1.08
MBCD25	10.11 ± 1.20
MBCD17	5.31 ± 1.10
MBCDF	11.45 ± 1.13
MBCD4	8.17 ± 1.14

In order to analyze for differences causing MTF resistance among these breast cancer cell lines, the status of EMT markers was measured. Interestingly, we found that MBCDF-D5, MBCD3, MBCD23, and MBCDF-B3 cells exhibited features of mesenchymal phenotype as disclosed by the lack of E-cadherin and presence of Vimentin and SNAIL, while MBCD25, MBCD17, MBCDF and MBCD4 cells expressed of E-cadherin with a concomitant absence of Vimentin and SNAIL, both distinctive of the epithelial phenotype (Fig. 1b). These data indicated that the response of primary breast cancer cell cultures to MTF exposure varied depending on the EMT status.

### Metformin decreases mesenchymal markers

**Fig. 2:**
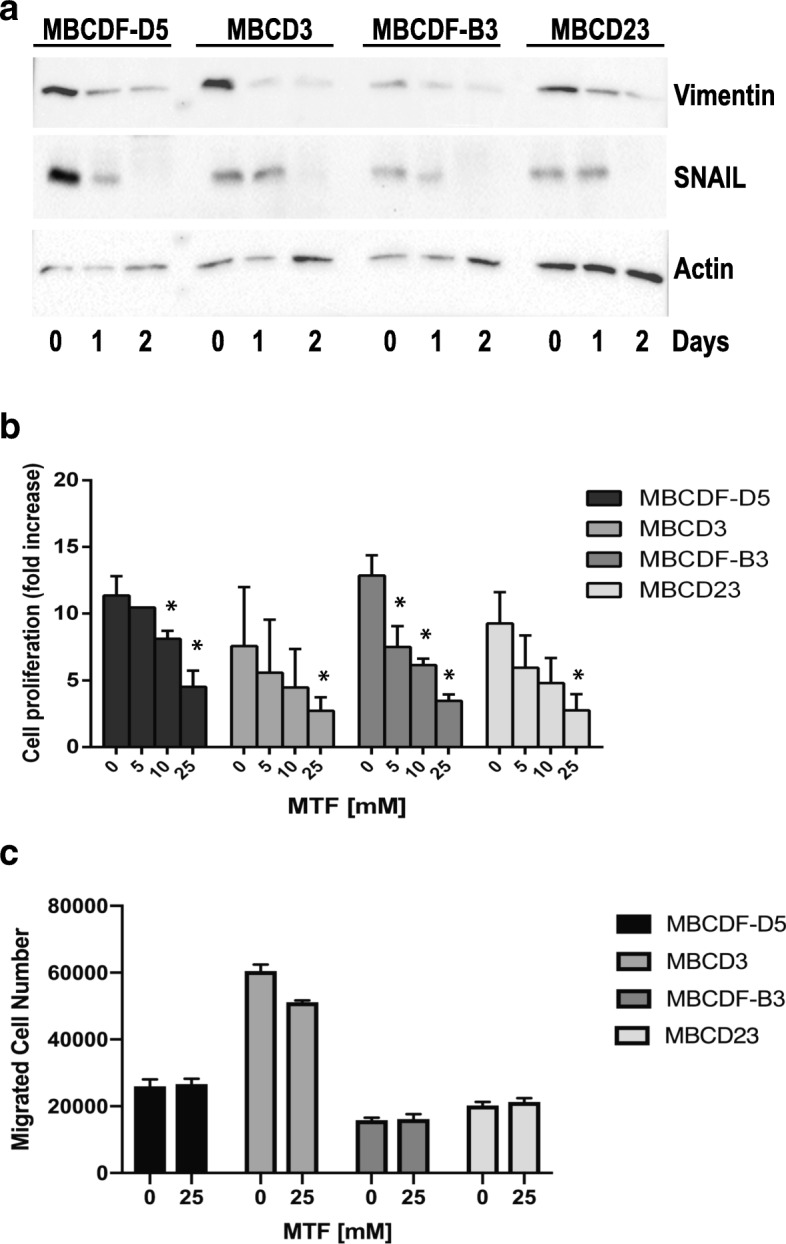
*Metformin reduces the expression of Vimentin and SNAIL, decreases cell proliferation but not migration in mesenchymal breast cancer cells.* **a** Primary breast cancer cell with mesenchymal phenotype (MBCDF-D5, MBCD3, MBCDF-B3 and MBCD23) were treated with 10 mM MTF for 0, 1 and 2 days and the effect of MTF on the mesenchymal markers Vimentin and SNAIL was analyzed by immunoblotting. Actin was used as loading control. **b** For cell proliferation, primary breast cancer cells with mesenchymal phenotype (MBCDF-D5, MBCD3, MBCDF-B3 and MBCD23) were seeded at 2500 cell/cm^2^ (5000 cells/well) in a 24 well-plate and incubated in the absence (control) or presence of 0, 5,10, and 25 mM MTF. Cell proliferation was evaluated by MTT at days 0, and 6. Data represents the mean ± SEM of three independent experiments performed in triplicate incubations. *P < 0.05. **c** Migration assays were performed using Boyden chambers. Thirty thousand cells with mesenchymal phenotype (MBCDF-D5, MBCD3, MBCDF-B3 and MBCD23) were seeded in the upper chamber in presence of MTF 0, 10 and 25 mM, the same concentrations of MTF were added in the in-bottom chamber, and then incubated for 6 h at 37 °C. After this time the cells that did not migrate were removed from the upper chamber. Cells that migrated were fixed and stained with Cristal Violet. Five fields were counted under the microscope at 20X. Migration assays were performed three independent times in triplicate

Several studies have suggested that MTF reverses EMT in several types of cancer [23, 24]. With this information in mind, we examined whether MTF affected the mesenchymal markers in MBCDF-D5, MBCD3, MBCD23, and MBCDF-B3 primary breast cancer cells. Cells were treated with 10 mM MTF for 24 and 48 h, and expression of Vimentin and SNAIL was analyzed by Western blot. The results showed that MTF treatment reduced the amount of Vimentin and SNAIL in a time-dependent manner (Fig. 2a). To examine the potential role of MTF on cell proliferation and migration of mesenchymal primary breast cancer cells, we performed cell proliferation assays in presence of MTF 0, 5, 10, and 25 mM. The effect of MTF was evaluated at day 6 by MTT assay (Fig. 2b). MTF reduced proliferation in a dose-dependent manner. The basal cell proliferation rate in these cells fluctuated between 7 and 12-fold. We observed that MTF 5 mM had no significant impact on any of this type of breast cancer cells. However, MTF at 10 and 25 mM had a major effect on cell proliferation, being MTF 25 mM where it was more significant (Fig. 2b, Additional file 2). Next, mesenchymal breast cancer cells (MBCDF-D5, MBCD3, MBCDF-B3 and MBCD23) treated either with 10 or 25 mM MTF for 6 h were used to evaluate cell migration by Boyden chamber assay (Fig. 2c). We found that cell migration was not affected by MTF at any of the two concentrations used.

### IL-6-induced epithelial-mesenchymal transition

**Fig. 3:**
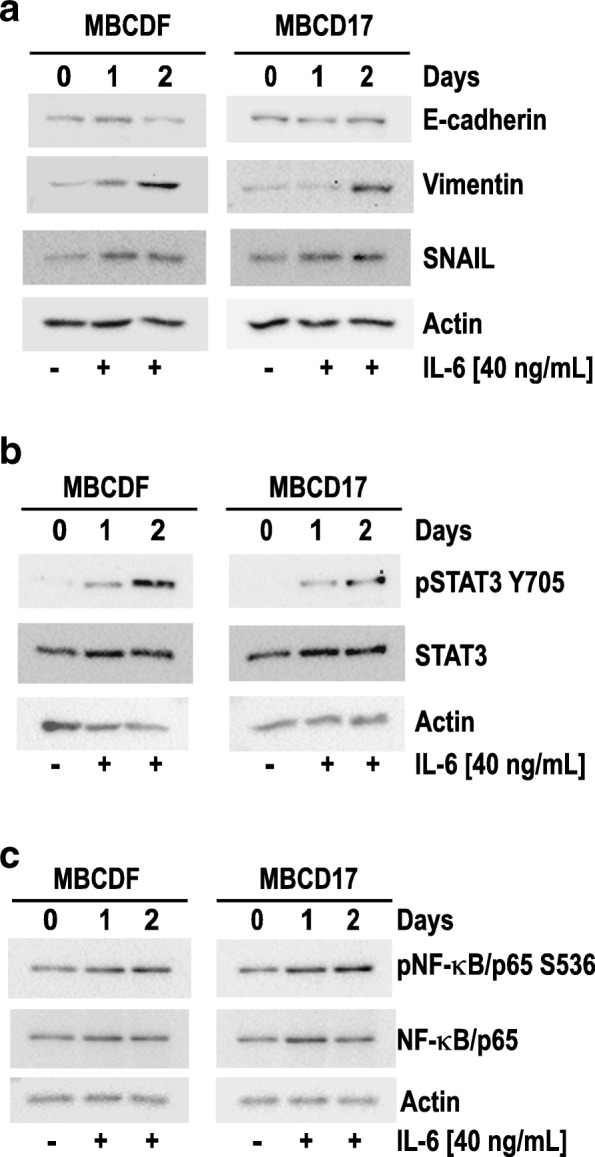
*Primary epithelial breast cancer cells undergo IL-6-induced EMT through STAT3 and NF-κB activation.* Primary breast cancer cells with epithelial phenotype (MBCDF and MBCD17) were treated with 40 ng/mL of IL-6 during 0, 1 and 2 days. **a** Induction of EMT was analyzed by assessing the expression of E-cadherin, Vimentin, and SNAIL by Western blots. **b** The activation of STAT3 was measured by phosphorylation of STAT3 on tyrosine 705 using a phospho-specific anti-pSTAT3 Y705 antibody. **c** Activation of NF-κB was assessed by analyzing phosphorylation of NF-κB/p65 on serine 536 employing a phospho-specific anti- pNF-κB S536 antibody. Actin was used as loading control in all cases

Since MTF down-regulated Vimentin and SNAIL levels in mesenchymal breast cancer primary cells, a model of EMT induction using IL-6, which is a well-known EMT inducer in several types of tumors including breast cancer [34, 35], was established. MBCDF and MBCD17 cells were treated with 40 ng/mL IL-6 for 1 and 2 days. A slight decrease in E-cadherin expression and an increase in Vimentin and SNAIL were concomitantly observed (Fig. 3a). Further, examination of two IL-6-induced transcription factors (STAT3 and NF-κB) revealed that IL-6 transactivated STAT3 as shown by the presence of increased STAT3Y705 phosphorylation and a slight increase in the total amount of STAT3 in a time-dependent fashion (Fig. 3b). Moreover, we found that NF-κB phosphorylation at S536 also was increased in response to IL-6 stimulation (Fig. 3c). These results indicate that the particular primary breast cancer cells studied can be induced to EMT by IL-6 exposure through the activation of STAT3 and NF-κB signaling pathways.

### Metformin reverses IL-6-induced epithelial mesenchymal transition

**Fig. 4:**
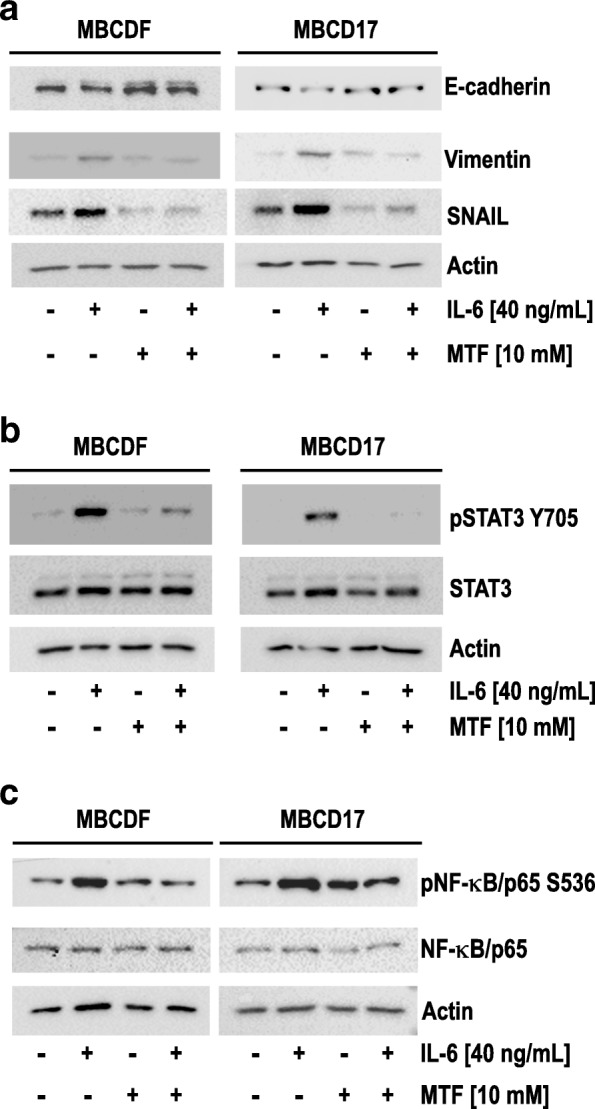
*Metformin reverses IL-6-induced EMT in primary epithelial breast cancer cells by inhibiting STAT3 and NF-κB phosphorylation.* MBCDF and MBCD17 cells were treated with 40 ng/mL IL-6. At day 1, MTF was added to cells incubated in the presence or absence of IL-6. After 2 days of incubation all conditions were collected for protein extraction and Western blot analysis. **a** Effect of MTF on IL-6-induced EMT markers (E-cadherin, Vimentin, and SNAIL). **b** Effect of MTF on IL-6-induced activation of STAT3 was assessed as in Fig. 3b. **c** Effect of MTF on IL-6-induced NF-κB phosphorylation was assessed as in Fig. 3c. Actin was used as loading control

Once an IL-6-induced EMT model in primary breast cancer cells was established, we investigated whether MTF is able to reverse EMT. MBCDF and MBCD17 primary epithelial breast cancer cells were treated with 40 ng/mL IL-6; after 24 h of IL-6 exposure, 10 mM MTF was added and cells were incubated for an additional 24 h period. As shown in Fig. 4a, IL-6 promoted EMT through lowering E-cadherin and increasing Vimentin and SNAIL. MTF alone did not exhibit a significant effect on EMT markers, while the addition of MTF to IL-6 treatment provoked re-expression of E-cadherin and inhibition of IL-6-stimulated Vimentin and SNAIL expression. These results indicate that MTF reverses the EMT induced by IL-6 in primary breast cancer cells.

We next examined the effect of MTF on the activation of IL-6-induced STAT3 and NF-κB in MBCDF and MBCD17 primary breast cancer cells. Similar experiments to those shown in Fig. 4a were performed and activation of the STAT3 and NF-κB pathways was analyzed. As shown in Fig. 4b, IL-6 induced phosphorylation of Y705 on STAT3 whereas MTF alone had no effect on STAT3 activation. However, addition of MTF to IL-6 stimulation reversed the phosphorylation of STAT3 at Y705 (Fig. 4b). In addition, IL-6 provoked phosphorylation of NF-κB at S536 (Fig. 3c), and reversed this phosphorylation when MTF was combined with IL-6 (Fig. 4c). Similar results were observed in both MBCDF and MBCD17 primary breast cancer cell cultures. These data suggest that MTF reverses EMT by blocking activation of the IL-6-induced transcription factors STAT3 and NF-κB.

### AMPK activation is required for decrease of pSTAT3, but not pNF-κB

**Fig. 5:**
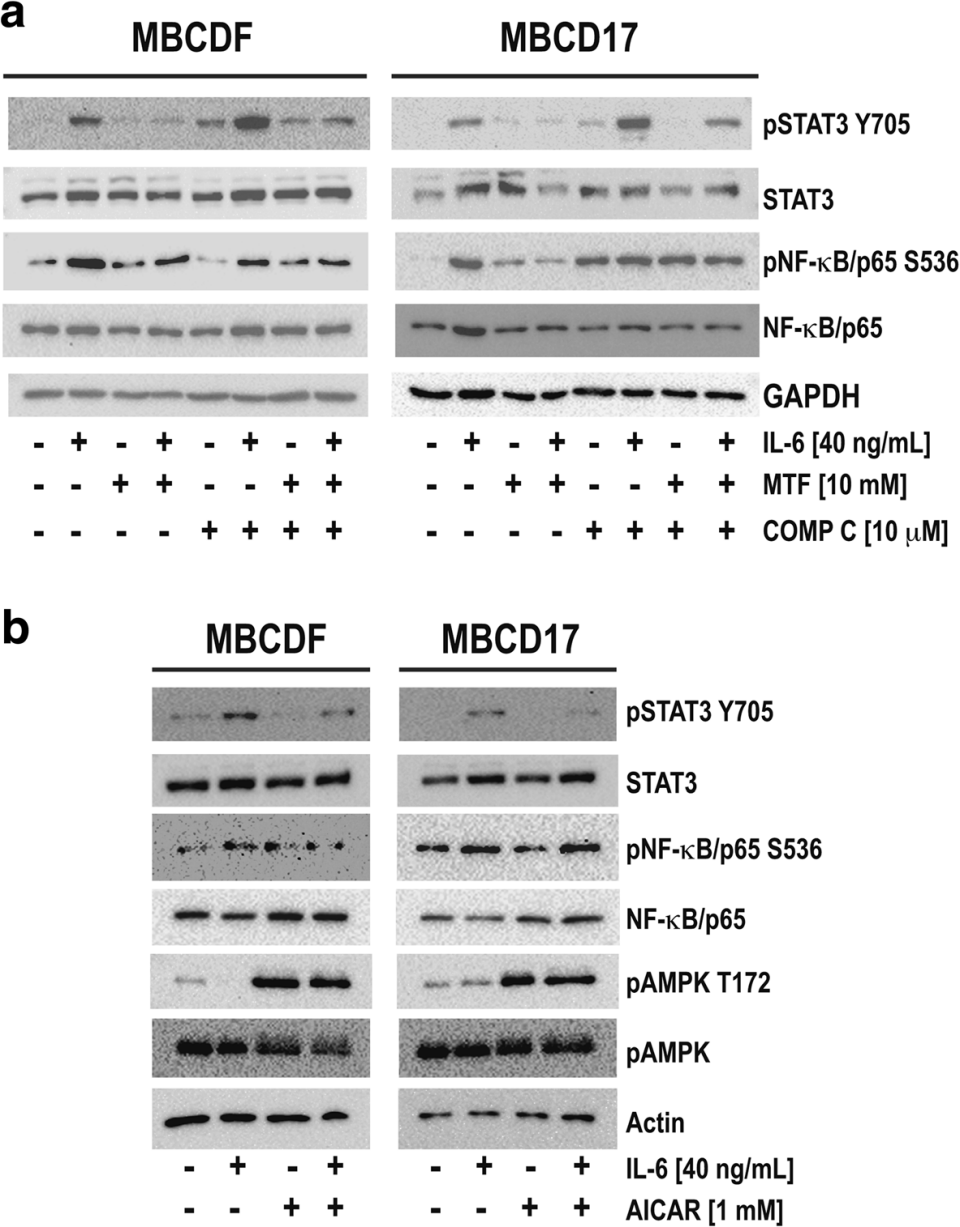
*Metformin effects on primary epithelial breast cancer cells are dependent of AMPK activation.* **a** MBCDF and MBCD17 primary breast cancer cell lines were treated with 40 ng/mL IL-6. At day 1, MTF was added to cells incubated with or without IL-6. Same experiment was repeated in the presence of 10 μM Compound C (COMP C) that was added 2 h before IL-6. The activation of STAT3 and NF-κB was measured by phospho-specific antibodies anti-pSTAT3 Y705 and anti-pNF-κB S536 antibody respectively. **b** MBCDF and MBCD17 cells were treated with 40 ng/mL IL-6, previous addition of 1 mM 5-aminoimidazole-4-carboxamide-1-β-D-ribofuranoside (AICAR), an activator of AMPK kinase. The activation of STAT3, NF-κB was evaluated as in Fig. 5a. AMPK activation was measured by a phospho-specific anti-pAMPK-T172

Several reports have shown that MTF anticancer effects may be dependent- or independent of AMPK [36]. In order to determine the role of AMPK in MTF-reduction of STAT3 and NF-κB phosphorylation in MBCDF and MBCD17 cells, we used two different approaches; AMPK inhibition with compound C (Dorsomorphin), or AMPK activation using an activator, 5-aminoimidazole-4-carboxamide-1-β-D-ribofuranoside (AICAR). For AMPK inhibition, 10 μM compound C was added alone or 2 h before IL-6 addition and incubated for 24 h. After this time, compound C alone and compound C + IL-6 conditions both were treated with MTF, incubation was extended further 24 h. Activation of STAT3 and NF-κB was evaluated as in Fig. 4. We observed that MTF reduced phosphorylation of both STAT3 and NF-κB as demonstrated before. Compound C alone did not have a significant effect on STAT3 phosphorylation (Fig. 5a, lane 5). Compound C added before IL-6 increased STAT3 phosphorylation (Fig. 5a, lane 6). Combination of compound C + MTF did not affect pSTAT3 Y705 (Fig. 5a, line 7) and treatment with compound C + IL-6 + MTF partially prevented the reduction of pSTAT3 Y705 (Fig. 5a, lane 8). These results suggest that AMPK inhibition with compound C partially interferes with the MTF-reduced STAT3 activation. In the case of NF-κB, we observed again that IL-6 induced NF-κB phosphorylation whereas co-treatment with MTF reduced IL-6-induced phosphorylation. Compound C alone exhibited opposite effects on pNF-κB S536, in MBCD17 increased phosphorylation while in MBCDF had no effect (Fig. 5a, lane 5). Both IL-6 + compound C and the combination of compound C + IL-6 + MTF presented similar levels of pNF-κBS536 similar to IL-6 treatment. These data suggest that the reduction in NF-κB activation induced by MTF is not dependent on AMPK.

Next, we examined whether AICAR-induced AMPK activation could mimic MTF reduction of IL-6-induced phosphorylation of STAT3 and NF-κB in MBCDF and MBCD17 breast cancer cells. Breast cancer cells treated with 1 mM AICAR alone or added 2 h before IL-6 were collected for protein extraction 2 days after treatment. We analyzed phosphorylation of STAT3 Y705 and NF-κB S536 (Fig. 5b). IL-6 induced phosphorylation of STAT3 Y705 whereas AICAR alone did not affect this phosphorylation; but when it was added before IL-6, IL-6-induced pSTAT3 Y705 was reduced (Fig. 5b). Next, we evaluated the effect of AICAR on the IL-6-induced NF-κB phosphorylation. We found that AICAR did not interfere with IL-6-induced phosphorylation of NF-κB. These data suggest that activation of AMPK can mimic reduction of pSTAT3 Y705 similar to that observed with MTF + IL-6. However, IL-6-induced pNF-κB S536 was not affected by AICAR (Fig. 5b). We confirm that AICAR induced AMPK activation by phosphorylation on T142 that indeed was increased by treatment (Fig. 5b). Together these results suggest that MTF-reduced phosphorylation of STAT3, but not NF-κB phosphorylation is dependent on AMPK activation.

### Metformin inhibits IL-6-induced cell proliferation and cell migration

**Fig. 6:**
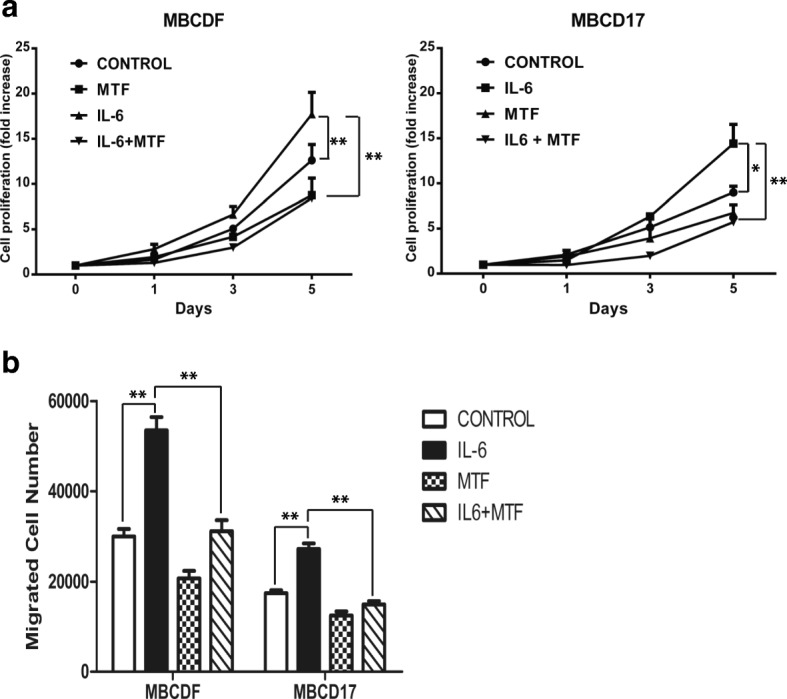
*Metformin inhibits IL-6-induced cell proliferation and migration*. **a** MBCDF and MBCD17 primary breast cancer cell lines were seeded at 15000 cells/cm^2^ in a 24-well plate and incubated under the absence (control) or presence of 10 ng/mL IL-6, 10 mM MTF or the combination of IL-6 and MTF. Cell proliferation was studied at days 0, 1, 3, and 5 by MTT. Data represent the mean ± SEM of three independent experiments performed in triplicate incubations. *P < 0.05, **P < 0.001. **b** Migration assays were tested using Boyden chambers. MBCDF and MBCD17 were seeded at 30000 cells/transwell in triplicate in the upper chamber. In the bottom chamber the same conditions were maintained as in Fig. 5a. Cells were allowed to migrate for 6 h. Migrating cells were fixed and stained with crystal violet. Data are presented as the mean ± SEM of three independent experiments. **P < 0.001

Since MTF interfered with IL-6-induced EMT of primary breast cancer cells, we then analyzed whether MTF had an effect on cell proliferation and migration. MBCDF and MBCD17 breast cancer cells were treated with IL-6 and MTF alone or in combination and cell proliferation was assessed by MTT assay at 0, 1, 3 and 5 days of stimulation. The basal rate of proliferation for MBCDF and MBCD17 cells reached 13- and 9-fold on day 6 respectively. IL-6 exposure increased cell proliferation up to 18-fold in MBCDF cells and 14-fold in MBCD17 cells. MBCDF and MBCD17 cells treated with MTF or with both IL-6 plus MTF showed a trend towards less proliferation than control cells, suggesting an inhibitory effect of MTF on IL-6-induced cell proliferation (Fig. 6a, Additional file 3). We next investigated the effect of MTF on IL-6-induced cell migration employing the Boyden chamber assay. The basal cell migration in the primary breast cancer cells studied showed different patterns, with MBCDF cells migrating more than MBCD17 cells. IL-6 treatment increased basal cell migration of both MBCDF and MBCD17 cells, whereas MTF-treated cells showed a downward trend migration when compared with control, unexposed cells. Migration in the presence of both IL-6 and MTF was similar to that exhibited by the control cells (Fig. 6b), suggesting that MTF interferes with the migration stimulated by IL-6.

## Discussion

In the present study, we analyzed the effects of MTF on the mesenchymal phenotype and IL-6-induced EMT in cultured primary breast cancer cells. EMT is a key process in metastasis development and the major cause of mortality among breast cancer patients and evidence has been accumulated over the past decade suggesting a potential role of MTF in suppressing the progression of several types of cancer [37]. We here demonstrate that MTF displays different effects associated with the EMT status of cultured primary breast cancer cells. Mesenchymal cells were resistant to MTF and epithelial cells were sensitive to MTF. Further analysis showed that high MTF doses reduced expression of mesenchymal markers as well as IL-6-induced EMT by blocking STAT3 and NF-κB phosphorylation. Reduction of STAT3 phosphorylation, but not that of NF-κB is dependent on AMPK activation. Additionally, MTF inhibited cell proliferation of mesenchymal breast cancer cells, but not cell migration. Moreover, MTF overturned IL-6-stimulated cell proliferation and migration of cultured primary breast cancer cells.

A number of studies have suggested a potential role of MTF on the prevention and improvement of overall survival in breast cancer [38, 39], and proposed potential mechanisms on how MTF may affect cell survival, proliferation, migration, and inflammation [40–42]. Many of these studies have been performed using immortalized breast cancer cell lines, which have been the standard experimental paradigm employed for many years. Nevertheless, cell lines may present several drawbacks including the effects of long time in culture on the potential development of new mutations and phenotypes [43, 44]. Thus, they frequently do not fully reflect what actually occurs in in vivo conditions*.* In this and other studies, we have used a model of cultured primary breast cancer cells that retain most of the biochemical features of the original tumor [32, 33]. Using this experimental model, we here demonstrate that the sensitivity to MTF depends on the EMT status: a mesenchymal phenotype correlated with resistance to MTF, whereas on the contrary, an epithelial phenotype was associated with sensitivity to MTF. In fact, differences in the IC50 for MTF indicated that mesenchymal cells required 4 to 10 times more MTF than epithelial cells to decrease 50% cell viability. Nonetheless, other studies have shown different effects of MTF on breast cancer cell lines. One study showed that MTF induced cell cycle arrest in estrogen receptor-positive but not in estrogen receptor-negative cells [45], while another study found that cells without expression of hormonal receptors were more responsive to this drug [46–48]. These studies proposed that differences in the response to MTF may be associated with particular breast cancer molecular subtypes [41, 47–49]. Here we demonstrate that the response to MTF in primary breast cancer cells is associated with the EMT status rather than with the molecular subtype.

During the EMT, cancer cells go through biochemical and morphological changes that allow them to acquire and enhance their invasive capacity [7]. We here show that Vimentin, SNAIL and cell proliferation decreased by MTF treatment in breast cancer cells with a mesenchymal phenotype, although these particular cells required higher doses of MTF to provoke an inhibitory effect. SNAIL expression has been associated with the repression of E-cadherin, invasion and metastases in several types of malignancies like breast, lung, hepatocellular and ovarian carcinomas [50–52]. SNAIL also has been associated as negative regulator of cell growth in lung and prostate cancer [53, 54]. Our results of the MTF-treated mesenchymal breast cancer cell, the reduction of SNAIL expression correlates with decreasing of cell proliferation. Consequently, MTF might reduce the invasive capacity of mesenchymal primary breast cancer cells by lowering SNAIL and Vimentin, which are also important factors involved in the structural changes of the cytoskeleton and thus in cell motility and invasiveness creating a phenotypic switch [55]. In fact, several studies have found that MTF represses EMT in several tumors including cervical cancer cells [56], thyroid cancer cells [57], hepatocellular carcinoma [58] and lung adenocarcinoma [25] by reducing the levels of these factors.

It has been shown that resumption of EMT promoted by growth factors and pro-inflammatory cytokines present in the tumor microenvironment is closely linked to this epithelial cell transformation and the acquisition of a metastatic phenotype [59, 60]. Factors involved in EMT in cancer include TNF, IL-1, and IL-4, which in turn activate several transcription factors that promote EMT [59]. Zinc finger protein SNAI1 or SNAIL is one of the transcription factors that regulate EMT and whose expression is governed by STAT3 [18]. In the present study, we tested whether primary cultures of breast cancer epithelial cells develop EMT when exposed to IL-6, a well-known pro-inflammatory cytokine that promotes EMT in several cancers via the JAK-STAT3-SNAIL signaling pathway [16–19]. We found that in cells exposed to IL-6, levels of Vimentin and SNAIL increased, albeit the changes observed in E-cadherin were subtle when compared to those previously detected in cell lines derived from lobular breast cancer tumors [61]. Nevertheless, our results correlate with previous studies in triple negative breast cancer cells, in which EMT induction was not associated to E-cadherin loss; in these particular cells, loss of E-cadherin expression was apparently an event occurring after the morphological changes promoted by EMT [61].

In addition to analyzing changes in biomarkers of EMT, we studied the activation of two transcription factors, STAT3 and NF-κB, both closely linked to EMT and activated by IL-6 [19, 62, 63]. These transcription factors, which regulate expression of Vimentin and SNAIL, increased in cultured primary breast cancer cells in response to IL-6. In this setting, we then explored the effects of MTF on IL-6-induced EMT. We found that MTF reduced IL-6-promoted upregulation of Vimentin and SNAIL allowing, in parallel, the recovering of E-cadherin levels from the subtle downregulation provoked by IL-6 exposure. Further, MTF also prevented IL-6-stimulated STAT3 and NF-κB phosphorylation. Concurrently, these data indicate that MTF inhibits EMT promoted by IL-6 by inhibiting STAT3 and NF-κB signaling, thereby reversing the cells to a less mesenchymal and invasive phenotype. Anticancer activities of MTF have been associated with activation of AMPK in a dependent or independent manner. AMPK is an energy sensor that is activated by several types of stress such as hypoxia, low glucose levels, oxidative stress, etc. [27]. On the other hand, AMPK has been described as a negative regulator of inflammatory response to IL-1, IL-6 and TNF [64]. We explored the putative role of AMPK in MTF-induced reduction of STAT3 and NF-κB phosphorylation. Our results show that inhibition of AMPK by using compound C blocks the inhibition of STAT3 phosphorylation provoked by MTF. We use another approach that consisted in the activation of AMPK by AICAR trying to mimic the effect of MTF; indeed, we observe that pre-treatment with AICAR before IL-6 reduces phosphorylation of STAT3. These data suggest that reduction of phosphorylation of STAT3 is mediated by AMPK. However, neither inhibition nor activation of AMPK affected MTF-mediated reduction of NF-κB phosphorylation.

It is also known that IL-6 participates in the regulation of migration and invasiveness of several types of cancer cells [15], including nasopharyngeal carcinoma cells, in which blocking of the IL-6 receptor by a specific monoclonal antibody reversed both processes and also EMT [65]. The effects of MTF on the proliferation and migration of cell lines derived from fibrosarcoma as well as from carcinomas of the thyroid, prostate, and pancreas also have been reported [57, 66, 67]. Considering this information, we explored the effects of this drug on the proliferation and migration of breast cancer cells with an initial epithelial phenotype and that were transformed to a mesenchymal phenotype by the exposure to IL-6. We found that MTF consistently inhibited both proliferation and migration of these cells, most probably by the reduction of IL-6-induced SNAIL and by antagonizing the effects of IL-6 on STAT3 and NF-κB phosphorylation. These results are in line with data from studies in cholangiocarcinoma cells suggesting that MTF inhibits migration and invasion through inactivation of the STAT3-mediated signaling pathway [68]. Our study additionally demonstrated that NF-κB activation may also be affected by MTF.

## Conclusions

In summary, the data presented herein indicate that the inhibitory effect of MTF on primary breast cancer cells depends on the EMT status. MTF efficiently decreases Vimentin, SNAIL and cell proliferation in mesenchymal breast cancer cells and also reverses IL-6-induced EMT by blocking STAT3 and NF-κB phosphorylation. Inhibition of STAT3 activation depends on AMPK activity. Further, MTF inhibits cell proliferation and cell migration induced by IL-6. These data suggest that MTF may represent a useful therapeutic strategy to reverse the metastatic phenotype, supporting its potential application as an add-on treatment associated to chemotherapy in breast cancer patients.

## Additional files

Additional file 1: **Table S1.** Molecular classification of primary breast cancer cells. (PDF 21 kb)

Additional file 2: *Effect of MTF on primary breast cancer cells with mesenchymal phenotype.* Cell proliferation of primary breast cancer cells with mesenchymal phenotype (MBCDF-D5, MBCD3, MBCDF-B3 and MBCD23) was assessed in a 24 well plate, were 2500 cell/cm^2^ were seeded (5000 cells/well) and incubated under the absence (control) or presence 5, 10, and 25 mM of MTF for 6 days. Phase-contrast images show the density of cells in a representative field of the well at day 6. Magnification 10X. (PDF 96 kb)

Additional file 3: *Effect of MTF on primary breast cancer cells with epithelial phenotype incubated with IL-6 and MTF*. MBCDF and MBCD17 primary breast cancer cell lines were seeded at 15000 cells/cm^2^ in a 24-well plate and incubated under the absence (control) or presence of IL-6 10 ng/mL, MTF 10 mM or the combination of IL-6 and MTF. Phase-contrast images show the density of cells in a representative field of the well at days 0, 1, 3, and 5. Magnification 10X. (PDF 89 kb)

## Data Availability

The datasets used and/or analyzed during the current study are available from the corresponding author on reasonable request.

## Abbreviations

AMPK: Adenosine monophosphate protein kinase
EMT: Epithelial mesenchymal transition
ER: Estrogen receptor
HER2: Epidermal growth factor receptor 2
IL-1: Interleukin-1
IL-4: Interleukin-4
IL-6: Interleukin-6
MTF: Metformin
NF-κB: Nuclear factor-κB
PR: Progesterone receptor
STAT3: Signal transducer and activator of transcription 3
TNF: Tumor necrosis factor
