# Supplementary material for: Metformin reverses mesenchymal phenotype of primary breast cancer cells through STAT3/NF-κB pathways
Source: BMC Cancer. 2019 Jul 23;19:728. doi: 10.1186/s12885-019-5945-1 (PMC6651945; doi:10.1186/s12885-019-5945-1)
Supplement: Supplementary file 1 — Table S1. Molecular classification of primary breast cancer cells. (PDF 21 kb) [file 12885_2019_5945_MOESM1_ESM.pdf]

Supplementary Table 1. Molecular classification of primary breast cancer cells

Primary breast cancer cell culture	Molecular subtype	HER2 status	ER status	PR status
MBCDF-D5	HER2	+	-	-
MBCD3	HER2	+	-	-
MBCD23	HER2	++	-	-
MBCDF-B3	HER2	++	-	-
MBCD25	Luminal B	++	-	+
MBCD17	Luminal B	++	-	+
MBCDF	HER2	+++	-	-
MBCD4	HER2	+	-	-

+++ High expression; ++ Medium expression; + Low expression; - Negative expression.

HER2 Epidermal growth factor receptor 2
ER Estrogen receptor
PR Progesterone receptor
