# Supplementary material for: Metformin reverses mesenchymal phenotype of primary breast cancer cells through STAT3/NF-κB pathways
Source: BMC Cancer. 2019 Jul 23;19:728. doi: 10.1186/s12885-019-5945-1 (PMC6651945; doi:10.1186/s12885-019-5945-1)
Supplement: Supplementary file 2 — Effect of MTF on primary breast cancer cells with mesenchymal phenotype. Cell proliferation of primary breast cancer cells with mesenchymal phenotype (MBCDF-D5, MBCD3, MBCDF-B3 and MBCD23) was assessed in a 24 well plate, were 2500 cell/cm2 were seeded (5000 cells/well) and incubated under the absence (control) or presence 5, 10, and 25 mM of MTF for 6 days. Phase-contrast images show the density of cells in a representative field of the well at day 6. Magnification 10X. (PDF 96 kb) [file 12885_2019_5945_MOESM2_ESM.pdf]

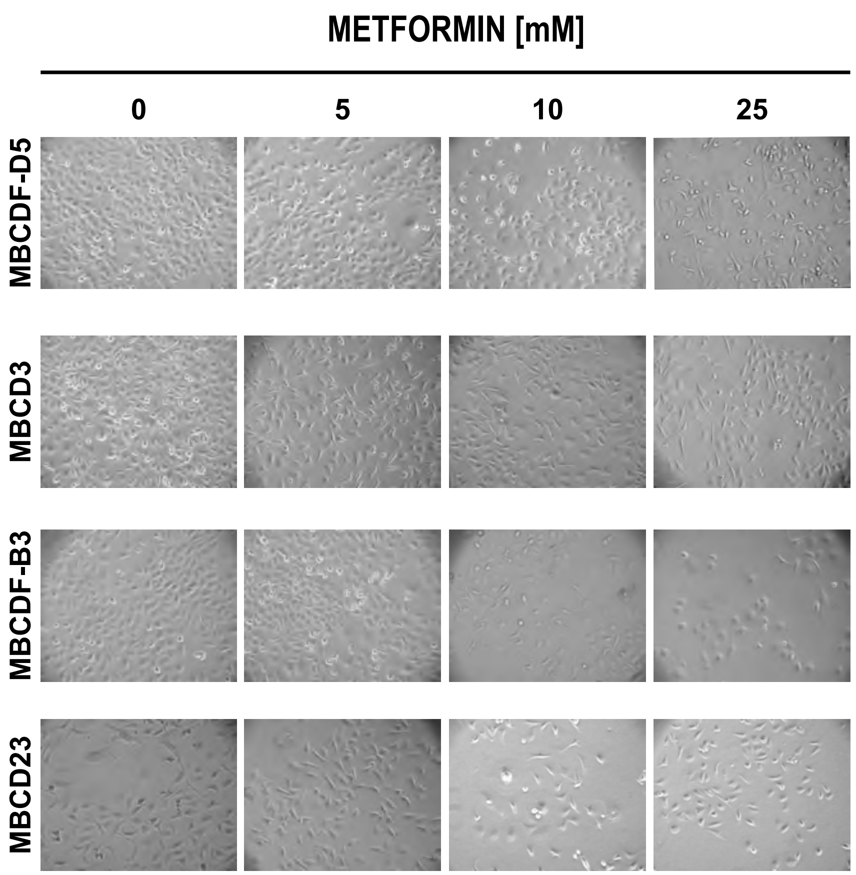

Additional file 2. *Effect of MTF on primary breast cancer cells with mesenchymal phenotype.* Cell proliferation of primary breast cancer cells with mesenchymal phenotype (MBCDF-D5, MBCD3, MBCDF-B3 and MBCD23) was assessed in a 24 well plate, were 2,500 cell/cm^2 were seeded (5,000 cells/well) and incubated under the absence (control) or presence 5, 10, and 25 mM of MTF for 6 days. Phase-contrast images show the density of cells in a representative field of the well at day 6. Magnification 10X.
