# Supplementary material for: Metformin reverses mesenchymal phenotype of primary breast cancer cells through STAT3/NF-κB pathways
Source: BMC Cancer. 2019 Jul 23;19:728. doi: 10.1186/s12885-019-5945-1 (PMC6651945; doi:10.1186/s12885-019-5945-1)
Supplement: Supplementary file 3 — Effect of MTF on primary breast cancer cells with epithelial phenotype incubated with IL-6 and MTF. MBCDF and MBCD17 primary breast cancer cell lines were seeded at 15000 cells/cm2 in a 24-well plate and incubated under the absence (control) or presence of IL-6 10 ng/mL, MTF 10 mM or the combination of IL-6 and MTF. Phase-contrast images show the density of cells in a representative field of the well at days 0, 1, 3, and 5. Magnification 10X. (PDF 89 kb) [file 12885_2019_5945_MOESM3_ESM.pdf]

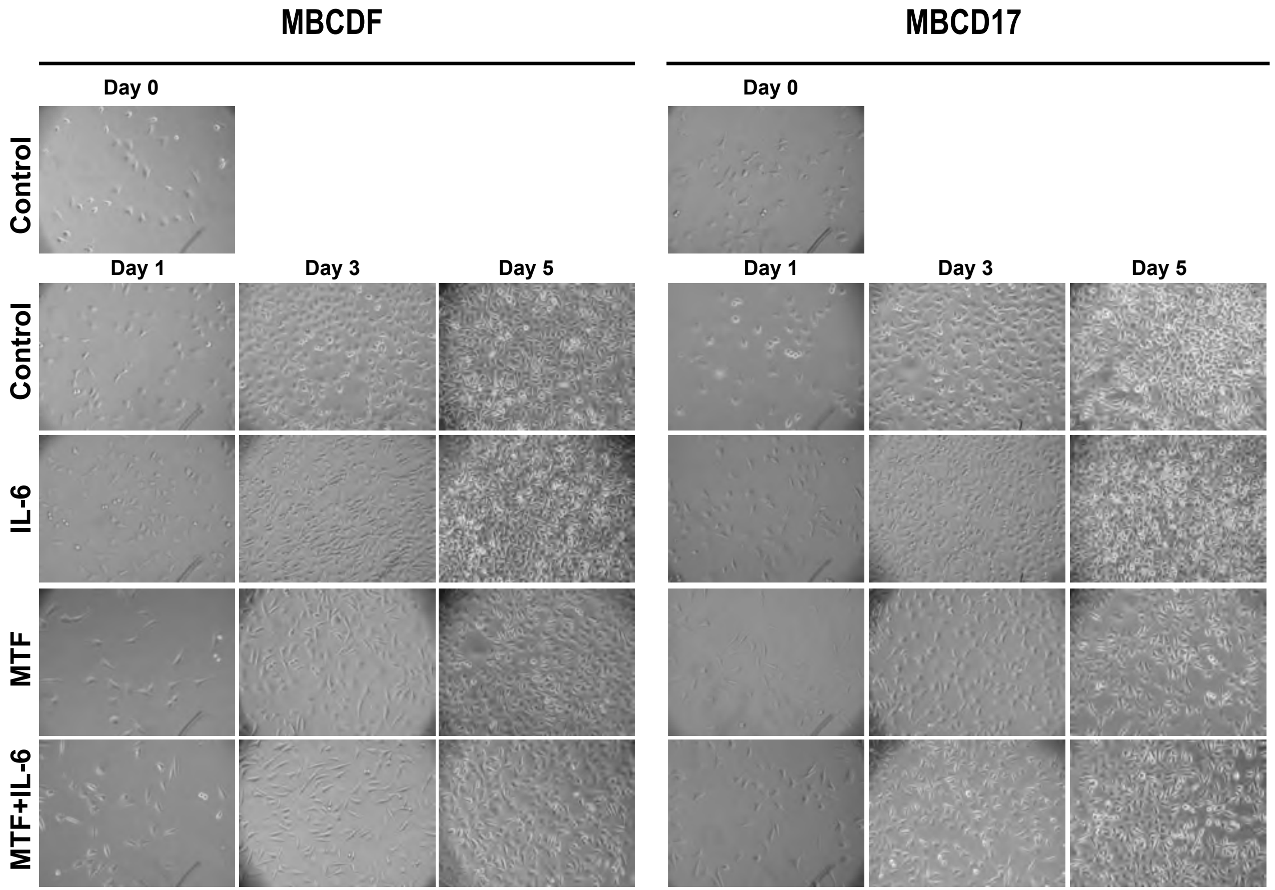

Additional file 3. *Effect of MTF on primary breast cancer cells with epithelial phenotype incubated with IL-6 and MTF*. MBCDF and MBCD17 primary breast cancer cell lines were seeded at 15 000 cells/cm^2 in a 24-well plate and incubated under the absence (control) or presence of IL-6 10 ng/mL, MTF 10 mM or the combination of IL-6 and MTF. Phase-contrast images show the density of cells in a representative field of the well at days 0, 1, 3, and 5. Magnification 10X.
